# Adaptive evolution of koala retrovirus transcription silencing and what it means for conservation

**DOI:** 10.1002/ctm2.70343

**Published:** 2025-07-15

**Authors:** Keith J. Chappell, Michaela B. J. Blyton, Zhiping Weng, William E. Theurkauf

**Affiliations:** ^1^ Australian Institute for Bioengineering and Nanotechnology, University of Queensland Brisbane Australia; ^2^ School of Chemistry and Molecular Biosciences, University of Queensland Brisbane Australia; ^3^ Australian Centre for Ecogenomics, School of Chemistry and Molecular Biosciences, University of Queensland Brisbane Australia; ^4^ Program in Molecular Medicine, University of Massachusetts Medical School Worcester Massachusetts USA

**Keywords:** chlamydia, conservation, Koala, piRNA, retrovirus

## Abstract

Koala populations in Australia face a barrage of threats, chiefly, habitat degradation and the effects of climate change including drought and bushfire. Further, high rates of chlamydiosis, linked to koala retrovirus (KoRV) viral load, is a major contributing factor to northern population decline. However, recent work by Yu et al., (Cell, 2024) has provided a glimmer of hope: some koalas have evolved ‘adaptive genome immunity’, which is able to actively suppress endogenous KoRV transcription. A single KoRV‐A provirus insertion within MAP4K4 gene's 3’ UTR is shown to be the trigger for production of sense and anti‐sense piRNAs, and that MAP4K4 KoRV integration is linked to both a 20% reduction in proviral genome integrations and 10‐fold reduction of KoRV transcription within male germline tissue. Here we discuss how this finding offers the potential to reduce koala disease burden and can be incorporated into conservation management to help save this iconic species.

Koala populations throughout much of their northern geographic range are facing catastrophic declines, such that they are now listed as *endangered* by the Australian Federal Government.^1^, ^2^ Koalas face a barrage of threats including habit loss, degradation and fragmentation due to land clearing and modification for housing and agriculture; vehicle impacts; domestic and feral animal attacks; as well as the impacts of climate change including prolonged droughts, heat waves and more frequent and intense bushfires. Disease is also a major contributing factor in these declines, with up to 85% of individuals infected with Chlamydia, causing infertility and/or mortality, in parts of the northern Australian state of Queensland.^3^ This high rate of the disease has been linked to a highly active koala retrovirus (KoRV) that is currently invading its genome.^4^

KoRV exists as both an infectious exogenous virus (transmitted between individuals) and as an endogenous retroviral element (integrated into the koala's germline DNA and passed vertically to progeny). KoRV is also the youngest known retrovirus to have invaded its host's germline.^5^ In comparison to endogenous retroviruses in all other species, KoRV is extremely active, as evidenced by the high level of insertion site polymorphisms,^5^ high circulating viral loads^6^ and tissue‐specific differences in pro‐viral copy numbers.^7^

While the innate and adaptive arms of the immune system provide a critical line of defence against pathogens, these become intractable once a retrovirus has integrated into a host's germline DNA as the virus is identified as “self”.^8^ Instead, all eukaryotes have evolved broad defences to limit the replication of endogenous retroviruses and other transposable elements (TEs). These germline integrations are controlled by PIWI‐interacting RNAs (piRNAs) that direct epigenetic modifications and degradation of transcribed sequences, leading to potent silencing of mobile genetic elements.^9^ While silencing of established TEs, involves both sense and anti‐sense piRNAs, our team's previous work identified only positive sense piRNAs against KoRV within koalas from south of the Queensland city of Brisbane.^10^ This work demonstrated that unspliced retroviral transcripts are processed into sense strand piRNAs, which was proposed to constitute a form of ‘innate genome immunity’ that could provide protection against endogenous retroviruses at the earliest stages of genome integration.^10^ However, this form of protection is ‘leaky’ and still allows some transcription of KoRV within the germline.

Incredibly, recent work by our team, Yu et al., (Cell, 2024), has identified a subpopulation of koalas, located just north of Brisbane that have evolved the ability to produce both sense and anti‐sense piRNAs, providing dual sense piRNA “adaptive genomic immunity”. These animals more effectively methylate KoRV genomic integrations and suppress KoRV transcription in the germline.^11^ Furthermore, the switch allowing for anti‐sense piRNA production was mapped to a single KoRV‐A proviral insertion within the 3′ UTR of the *MAPAK4* gene. The *MAP4K4* KoRV integration was linked to a 10‐fold reduction in KoRV transcription in testicular germline tissue in a comparison of six male koalas (*p* = 0.04).^11^

The majority of individual koalas share very few proviral integration sites,^10^, ^11^ however in stark contrast, 25 of 83 animals surveyed in the region north of the Brisbane River shared the *MAP4K4* KoRV integration along with two animals south of the Brisbane River.^11^ Comparison between the two populations revealed a 20% reduction in the total number of KoRV proviral integrations in animals north of the Brisbane River where the *MAP4K4* proviral integration is common (*p* = 2.2 × 10^−22^). These findings are highly suggestive of the *MAP4K4* proviral integration being under positive selection and expanding through the koala population.

The identification of the *MAP4K4* KoRV integration as a trigger for effective KoRV silencing and that positive selection is leading to increasing prevalence amongst koalas provides a distant glimmer of hope that the species can overcome the impacts of the endogenous retrovirus that is literally rewriting the koala genome. Fundamental mechanisms that protect against germline invasion should not be entirely unexpected given the high prevalence of ancient endogenous retroviral elements (ERVs) with eukaryotic genomes, including 8% of the human genome.^12^ However, the invasion of the koala genome is entirely unique, as it is the only known example in which this process is currently occurring and can be directly studied.

KoRV is not the only active retrovirus found within the genome of koalas. Three additional ERVs (KoERV.1, KoERVK.14 and KoERVL.1) show an extremely high degree of insertion site polymorphism. This level of ERV activity is highly unusual and well beyond that seen in any other species examined.^13^, ^14^, ^15^ To put this into perspective, an analysis of > 2500 human genomes revealed a total of just 36 polymorphic ERV insertion sites,^13^ whereas our analysis of 10 koalas has revealed around 1000 polymorphisms unique to individual animals. Given that in humans, individual insertion sites can be linked with cancer, autoimmunity and other detrimental effects,^16^ it is incredible that koalas can persist despite such extensive genomic rewriting.

While the findings of Yu et al., (Cell, 2024) are monumental, many unknowns remain. It is logical to expect that a reduction in KoRV germline translocation to be associated with reduced incidence of non‐viable embryos and lower risk of cancer, however, we do not know how the *MAP4K4* KoRV integration and dual sense piRNA silencing may impact KoRV activity in somatic cells and affect susceptibility to disease. Those koalas with the MAP4K4 insertion did not more effectively suppress KoRV transcription in the liver. However, reduced proviral copy numbers and germline methylation may yet prove important for disease susceptibility. Of note, a recent comparative assessment of two wild koala populations, north and south of the Brisbane River, revealed higher chlamydia disease in the southern subpopulation,^17^ of which likely lack the *MAP4K4* KoRV integration. However, further studies would need to be undertaken to confirm the specific role of the MAP4K4 insertion in protection from disease. Furthermore, as only a limited region within the koala's total geographic range has been investigated to date, we have no idea whether the *MAP4K4* KoRV integration is the only trigger for effective KoRV silencing. Other subpopulations may have acquired alternate genomic integrations that result in similar or potentially even more effective KoRV silencing. The relationship between KoRV and the other three highly active ERVs has only just begun to be understood. It is assumed that KoRV played a role in the reactivation of other ERVs, but the mechanism is unknown, and it is also unknown whether transcriptional silencing of KoRV by sense and anti‐sense piRNAs will in time lead to inactivation and fixation of these other active ERVs as well.

Above all, it should be emphasized that the germline immunity and positive selection of the *MAP4K4* KoRV integration works on an evolutionary timescale. The major threats faced by koalas, which have led to steep population declines are much more acute. Conservation efforts rightly need to focus on lessening the impacts of habit degradation, droughts and bushfires, but this does not mean the findings from Yu et al., (Cell, 2024) are irrelevant. In a recent publication, we demonstrated a strong association between KoRV load, the exogenous KoRV subtypes and chlamydial disease pathologies including conjunctivitis, cystitis, renal pathology and reproductive pathology, as well as, overall poor body condition.^4^ In the time since this publication, captive breeding programs have begun to implement processes such as screening for KoRV load and exogenous subtype presence. Similarly, breeding programs could integrate animals with the *MAP4K4* KoRV integration from north of Brisbane to accelerate the spread of adaptive germline immunity against KoRV‐A into new populations. We believe that in addition to the primary goal of these programs to re‐populate declining or extinct wild populations, there is the opportunity to breed more resilient koalas that are less prone to secondary infections, such as chlamydia, and better equipped to withstand the negative impacts of genetic rewriting by endogenous KoRV. Repopulation of areas where koala numbers are currently in steep decline with more resilient animals has the potential to reverse population declines and provides a glimmer of hope for this endangered and iconic species.

## AUTHOR CONTRIBUTIONS

All authors contributed to the writing of this editorial.

## ETHICS STATEMENT

The authors declare that data included in this editorial are correct and that the viewpoints are are their own.
